# Socioeconomic inequality in health care use among cancer patients in China: Evidence from the China health and retirement longitudinal study

**DOI:** 10.3389/fpubh.2022.942911

**Published:** 2022-08-02

**Authors:** Huiru Zhang, Yu Fu, Mingsheng Chen, Lei Si

**Affiliations:** ^1^School of Health Policy and Management, Nanjing Medical University, Nanjing, China; ^2^Center for Global Health, Nanjing Medical University, Nanjing, China; ^3^The George Institute for Global Health, UNSW Sydney, Kensington, NSW, Australia

**Keywords:** inequality, health care use, cancer patients, concentration index, China

## Abstract

**Background:**

Cancer is a major public health problem worldwide and the leading cause of death in China, with increasing incidence and mortality rates. This study sought to assess socioeconomic-related inequalities in health care use among cancer patients in China and to analyze factors associated with this disparity.

**Methods:**

This study used data collected for the China Health and Retirement Longitudinal Study in 2018. Patients who reported having cancer were included. The annual per capita household expenditure was classified into five groups by the quintile method. We calculated the distribution of actual, need-predicted, and need-standardized health care use across different socioeconomic groups among patients with cancer. The concentration index (CI) was used to evaluate inequalities in health care use. Influencing factors of inequalities were measured with the decomposition method.

**Results:**

A total of 392 people diagnosed with cancer were included in this study. The proportion of cancer patients who utilized outpatient and inpatient services was 23.47% and 40.82%, respectively, and the CIs for actual outpatient and inpatient service use were 0.1419 and 0.1960. The standardized CIs (CI for outpatient visits = 0.1549; CI for inpatient services = 0.1802) were also both positive, indicating that affluent cancer patients used more health services. The annual per capita household expenditure was the greatest factor favoring the better-off, which contributed as much as 78.99% and 83.92% to the inequality in outpatient and inpatient services use, followed by high school education (26.49% for outpatient services) and living in a rural village (34.53% for inpatient services). Urban Employee Basic Medical Insurance exacerbated the inequality in inpatient services (21.97%) while having a negative impact on outpatient visits (−22.19%).

**Conclusions:**

There is a pro-rich inequality in outpatient and inpatient services use among cancer patients in China. A lower socioeconomic status is negatively associated with cancer care use. Hence, more targeted financial protection for poor people would relieve cancer patients of the burden caused by the high cost of cancer care.

## Introduction

Cancer is a major public health problem globally and has become the leading cause of death and illness in China (1). The International Agency for Research on Cancer estimated that 19.3 million cancer cases were newly diagnosed worldwide and nearly 10.0 million cancer-related deaths occurred in 2020 (2). With a rapidly aging population worldwide and an increase in unhealthy lifestyles, cancer has been identified as the primary cause of death, reducing the survival time of cancer patients (3). An estimated 4.6 million new cancer cases and 3.0 million cancer deaths occurred in China in 2020 (4). China had a slightly lower cancer incidence rate but substantially higher cancer mortality compared to other countries (5).

Developing countries accounted for >56% of the total new annual incidence of cancer patients, with a total cancer-related mortality rate of 64% (6). Lung and bronchus cancer was commonly diagnosed and identified as the leading cancer killer in China, with ~781,000 new cases and 626,000 deaths every year, followed by stomach, esophageal, liver and colorectum cancers (7). In 2015, the mortality attributed to these five types of cancer accounted for about three-quarters of all cancer mortality (5). In addition, 16.6% of the total disease burden (measured in DALYs) were attributed to cancer in China (8). Meanwhile, studies have found that cancer patients often bear considerable medical expenditure. The overall incidence rate of catastrophic health expenditure in cancer patient families was estimated at 60.0% (9, 10). Patients with cancer from socioeconomically disadvantaged households were particularly financially vulnerable due to the high costs of cancer care, which prevented them from accessing health care. Health care use by cancer patients in lower socioeconomic status groups was limited (11, 12). A systematic review based on cancer inequalities studies has concluded that there were statistically significant socioeconomic inequalities in cancer biological and precision therapy utilization, and a 1.2-fold gap in cancer therapies treatment between cancer patients with the lowest socioeconomic status and the highest socioeconomic status was observed (13). The rich cancer patients tended to use more health care. In addition, health care costs might be particularly challenging for those without health insurance who were more likely to pay greater out-of-pocket costs (14). Hence, the disparity in health care use in China remains a major issue to maximizing total health.

The inequality in health has been a major priority of the health system globally (15). Several studies have contributed an extensive amount of research on the many different dimensions of cancer outcome inequality (16–19), including reporting gradients in cancer incidence, mortality, and survival were associated with deprivation and lower socioeconomic status. However, socioeconomic inequalities in health care use or behavior among cancer patients remain largely unexplored, although this type of inequality has also been observed in some high-income countries, such as South Korea, Australia, and England (20–22).

Previous studies have highlighted systematic differences in cancer care use, with higher incidence rates and inadequate use being more prevalent in lower socioeconomic status groups. Moreover, income substantially affected the use of health care (23). However, existing research has only focused on the association between socioeconomic status and health care use inequalities among cancer patients; to date, the effects of other socioeconomic and need factors remain unclear. Furthermore, no systematic analysis of health care use inequality and influencing factors among cancer patients in China has been published. Hence, this study sought to close these gaps by measuring socioeconomic inequalities in health care use among patients with cancer in China in order to determine which areas will require more attention in the future.

## Methods

### Study design and data sources

This study was based on data collected from the China Health and Retirement Longitudinal Study in 2018, which was conducted by the China Center for Economic Research of Peking University. The survey used a questionnaire to collect data, such as demographic characteristics, socioeconomic status, social security level, and physical health status of patients. Using a multistage probability-proportional-to-size sampling, a total of 19,507 individuals aged ≥45 years were identified. Patients who were reported as having cancer and had no missing values for dependent variables were considered eligible for inclusion. After excluding those with missing relevant variables, a total of 392 individuals were finally included in this study.

### Socioeconomic status

The annual per capita household expenditure was adopted as a proxy for socioeconomic status (24) and used to group individuals into five groups, from the lowest to the highest. The quintile of socioeconomic status categories was determined within each county or district and then pooled across all sampled counties and districts because the level of economic development differed between sampling regions.

### Variables

#### Dependent variables

Two variables of health care use were employed. Patients with cancer were asked if they had visited a public hospital, private hospital, public health center, clinic, or health worker's or doctor's practice or been visited by a health worker or doctor for outpatient care in the last month (not including for a physical examination) and had they received inpatient care in the past year. The answers to these questions were coded as a dummy variable (0 = no, 1 = yes).

#### Independent and control variables

The following variables were included to investigate the relationship of socioeconomic status and health care use: gender (male or female), age (45–59, 60–74, or ≥75 years), educational level (primary school or below, middle school, or high school and above), marital status [single (separated/divorced/widowed/never married), married or partnered], employment status (unemployed, employed, or retired), impoverished status (no or yes), region (east, central, west, or northeast), Hukou type (agricultural Hukou or non-agricultural Hukou), region of residence (urban, suburban, or rural), health insurance [no health insurance, Urban Employee Basic Medical Insurance (UEBMI), Urban and Rural Resident Basic Medical Insurance (URRBMI), Urban Resident Basic Medical Insurance (URBMI), New Rural Cooperative Medical Scheme (NRCMS), or another], number of people in the household, physical examination (no or yes), self-reported health status (very good, good, fair, poor, or very poor), disability (no or yes), degree of pain (none, a little, somewhat, quite a bit, or very much), smoking (no or yes), and alcohol consumption (no or yes).

### Statistical analysis

#### Measurement of concentration index

The measurement of the CI proposed by Wagstaff et al. (25, 26) was used to examine the magnitude of socioeconomic inequality according to Equation 1.


(1) $$\begin{array}{r} C=\frac{2}{\mu }\text{cov}\left({\text{h}}_{\text{i}},{\text{r}}_{\text{i}}\right) \end{array}$$

where h_i_ is the measure of actual health service use, μ is its mean and r_i_ is the relative fractional rank of an individual i in the distribution of the annual per capita household expenditure (i = 1 for the lowest and i = n for the highest).

According to Wagstaff et al. (24), the CI is defined as twice the area between the concentration curve and the line of equality, where a concentration curve plots the cumulative proportion of the use of services (y-axis) against the cumulative percentage of respondents, ranked by the annual per capita household expenditure, beginning with the least affluent and ending with the most affluent (x-axis). The CI ranges from −1 to 1. When the concentration curve lies below the diagonal (45° line), the CI is a positive value, indicating the concentration of health inequality in favor of the rich (pro-rich) (27).

#### Analysis of decomposition method

The decomposition method proposed by Wagstaff et al. (28) was employed to measure factors associated with inequalities. They demonstrated that the health CI can be decomposed into the contributions of individual factors to income-related health inequality, in which each contribution is the product of the sensitivity of heath with respect to that factor and the degree of income-related inequality in that factor. A decomposition analysis estimates how determinants proportionally contribute to inequality in the use of services. A positive value of contribution to socioeconomic inequality means a positive association with health care use; in other words, the variable increases pro-rich inequality and outpatient or inpatient services is more concentrated in the richer population.

The overall inequality in health services use (C) is written as:


(2) $$\text{C}=\sum_{\text{j}}\left(\beta_{\text{j}}^{\text{m}}{\bar{\text{x}}}_{\text{j}}/\mu \right){\text{C}}_{\text{j}}+\sum_{\text{k}}\left(\gamma_{\text{k}}^{\text{m}}{\bar{\text{z}}}_{\text{k}}/\mu \right)\text{Ck+GC/}\mu$$

where μ is the mean of y, ${\bar{x}}_{\text{j}}$ is the mean of x_j_, C_j_ and C_k_ are the CI of need and non-need variables; and GC is the error term of health care.

All analyses were performed using SPSS version 25.0 (IBM Corp., Armonk, NY, USA) and Stata version 16.0 (Stata Corp., College Station, TX, USA). A two-sided value of 0.05 was considered to be statistically significant.

## Results

### Social demographic characteristics of cancer patients

A total of 392 cancer-related cases were observed, of which 23.47% had visited for outpatient care during the past month and 40.82% had received inpatient services in the last year. Cancer was most prevalent in male and female individuals aged 60–74 years (50.51%). About 2/3 (62.76%) of patients with cancer reported retirement and unemployment with their cancer diagnosis. Only 16 (4.08%) reported being uninsured. Of note, individuals from socioeconomically disadvantaged households were less likely to seek outpatient and inpatient services compared to better-off individuals. Other descriptive statistics of health care use and cancer patients' characteristics are shown in Table 1.

**Table 1:** Descriptive characteristics of cancer patients.

	**Outpatient visits**, ***n*** **(%)**		**Inpatient services**, ***n*** **(%)**		**Total *N* = 392**
	**No *n* = 300 (76.53%)**	**Yes *n* = 92 (23.47%)**	**No *n* = 232 (59.18%)**	**Yes *n* = 160 (40.82%)**	
**Gender**					
Male	117 (75.48%)	38 (24.52%)	81 (52.26%)	74 (47.74%)	155 (39.54%)
Female	183 (77.22%)	54 (22.78%)	151 (63.71%)	86 (36.29%)	237 (60.46%)
**Age, years**					
45–59	111 (78.72%)	30 (21.28%)	93 (65.96%)	48 (34.04%)	141 (35.97%)
60–74	149 (75.25%)	49 (24.75%)	112 (56.57%)	86 (43.43%)	198 (50.51%)
≥75	40 (75.47%)	13 (24.53%)	27 (50.94%)	26 (49.06%)	53 (13.52%)
**Educational level**					
Primary school or below	204 (77.57%)	59 (22.43%)	153 (58.17%)	110 (41.83%)	263 (67.09%)
Middle school	74 (79.57%)	19 (20.43%)	63 (67.74%)	30 (32.26%)	93 (23.72%)
High school and above	22 (61.11%)	14 (38.89%)	16 (44.44%)	20 (55.56%)	36 (9.18%)
**Employment status**					
Unemployed	157 (72.35%)	60 (27.65%)	113 (52.07%)	104 (47.93%)	217 (55.36%)
Employed	119 (81.51%)	27 (18.49%)	101 (69.18%)	45 (30.82%)	146 (37.24%)
Retired	24 (82.76%)	5 (17.24%)	18 (62.07%)	11 (37.93%)	29 (7.40%)
**Region**					
East	113 (76.87%)	34 (23.13%)	95 (64.63%)	52 (35.57%)	147 (37.50%)
Central	89 (76.72%)	27 (23.28%)	64 (55.17%)	52 (44.83%)	116 (29.59%)
West	79 (74.53%)	27 (25.47%)	59 (55.66%)	47 (44.34%)	106 (27.04%)
Northeast	19 (82.61%)	4 (17.39%)	14 (60.87%)	9 (39.13%)	23 (5.87%)
**Region of residence**					
Urban areas	86 (76.11%)	27 (23.89%)	56 (49.56%)	57 (50.44%)	113 (28.97%)
Suburban areas	27 (75.00%)	9 (25.00%)	21 (58.33%)	15 (41.67%)	36 (9.23%)
Rural village	185 (76.76%)	56 (23.24%)	154 (63.90%)	87 (36.10%)	241 (61.79%)
**Socioeconomic status**					
Quintile 1 (lowest)	26 (83.87%)	5 (16.13%)	22 (70.97%)	9 (29.03%)	31 (8.01%)
Quintile 2	81 (79.41%)	21 (20.59%)	68 (66.67%)	34 (33.33)	102 (26.36%)
Quintile 3	87 (80.56%)	21 (19.44%)	67 (62.04%)	41 (37.96%)	108 (27.91%)
Quintile 4	71 (71.72%)	28 (28.28%)	46 (46.46%)	53 (53.54%)	99 (25.58%)
Quintile 5 (highest)	32 (68.09%)	15 (31.91%)	25 (53.19%)	22 (46.81%)	47 (12.14%)
**Health insurance**					
No health insurance	10 (62.50%)	6(37.50%)	12 (75.00%)	4 (25.00)	16 (4.08%)
UEBMI	63 (74.12%)	22 (25.88%)	44 (51.76%)	41 (48.24%)	85 (21.68%)
URRBMI	34 (80.95%)	8 (19.05%)	27 (64.29%)	15 (35.71%)	42 (10.71%)
URBMI	14 (87.50%)	2 (12.50%)	11 (68.75%)	5 (31.25%)	16 (4.08%)
NRCMS	170 (76.23%)	53 (23.77%)	132 (59.19%)	91 (40.81%)	223 (56.89%)
Another†	9 (90.00%)	1 (10.00%)	6 (60.00%)	4 (40.00%)	10 (2.55%)
**Self-reported health status**					
Very good	14 (87.50%)	2 (12.50%)	12 (75.00%)	4 (25.00%)	16 (4.62%)
Good	20 (86.96%)	3 (13.04%)	17 (73.91%)	6 (26.09%)	23 (6.65%)
Fair	93 (78.15%)	26 (21.85%)	86 (72.27%)	33 (27.73%)	119 (34.39%)
Poor	97 (75.19%)	32 (24.81%)	67 (51.94%)	62 (48.06%)	129 (37.28%)
Very poor	40 (67.80%)	19 (32.20%)	29 (49.15%)	30 (50.85%)	59 (17.05%)
**Disability**					
No	168 (79.25%)	44 (20.75%)	135 (63.68%)	77 (36.32%)	212 (54.08%)
Yes	132 (73.33%)	48 (26.67%)	97 (53.89%)	83 (46.11%)	180 (45.92%)
**Pain degree**					
None	106 (82.81%)	22 (17.19%)	87 (67.97%)	41 (32.03%)	128 (32.65%)
A little	85 (77.27%)	25 (22.73%)	63 (57.27%)	47 (42.73%)	110 (28.06%)
Somewhat	44 (69.84%)	19 (30.16%)	35 (55.56%)	28 (44.44%)	63 (16.07%)
Quite a bit	29 (74.36%)	10 (25.64%)	19 (48.72%)	20 (51.28%)	39 (9.95%)
Very much	36 (69.23%)	16 (30.77%)	28 (53.85%)	24 (46.15%)	52 (13.27%)
**Smoking**					
No	193 (78.46%)	53 (21.54%)	157 (63.82%)	89 (36.18%)	246 (62.76%)
Yes	107 (73.29%)	39 (26.71%)	75 (51.37%)	71 (48.63%)	146 (37.24%)

† represents Government Employee Health Insurance.

UEBMI, Urban Employee Basic Medical Insurance; URRBMI, Urban and Rural Resident Basic Medical Insurance; URBMI, Urban Resident Basic Medical Insurance; NRCMS, New Rural Cooperative Medical Scheme.

### Distribution of health care use among patients with cancer

Table 2 presents the actual, need-predicted, and need-standardized distribution for outpatient and inpatient services use by cancer patients across socioeconomic status groups. CIs for inequality and concentration curves are also reported.

**Table 2:** Distribution of actual, need-expected, and need-standardized use of outpatient and inpatient services among cancer patients across different socioeconomic status groups.

**Socioeconomic status**	**Outpatient visits use**			**Inpatient services use**		
	**Actual**	**Need-Expected**	**Need-Standardized**	**Actual use**	**Need-Expected**	**Need-Standardized**
Quintile 1 (lowest)/%	20.47	22.28	19.85	29.92	36.56	31.16
Quintile 2/%	18.33	21.73	18.26	35.00	38.26	34.54
Quintile 3/%	27.63	21.39	27.90	47.37	39.15	46.02
Quintile 4/%	29.63	21.61	29.68	48.15	40.26	45.69
Quintile 5 (highest)/%	24.00	19.27	26.39	52.00	33.55	56.25
All/%	23.39	21.66	23.39	39.18	37.80	39.18
CI	0.1419**	−0.0140	0.1549**	0.1960**	0.0164	0.1802**

CI, concentration index.

* *p* < 0.05,

** *p* < 0.001.

The CIs for actual outpatient and inpatient services use were both positive, and the values of the indices for inpatient services were much higher than those for outpatient visits (CI for outpatient visits = 0.1419, *p* < 0.05; CI for inpatient services = 0.1960, *p* < 0.05). With regard to need-expected use, the CI was not statistically significant in both outpatient and inpatient services, and proportionality was not rejected in either case (CI for outpatient visits = −0.0140, *p* > 0.05; CI for inpatient services = 0.0164, *p* > 0.05).

**Figure 1:**
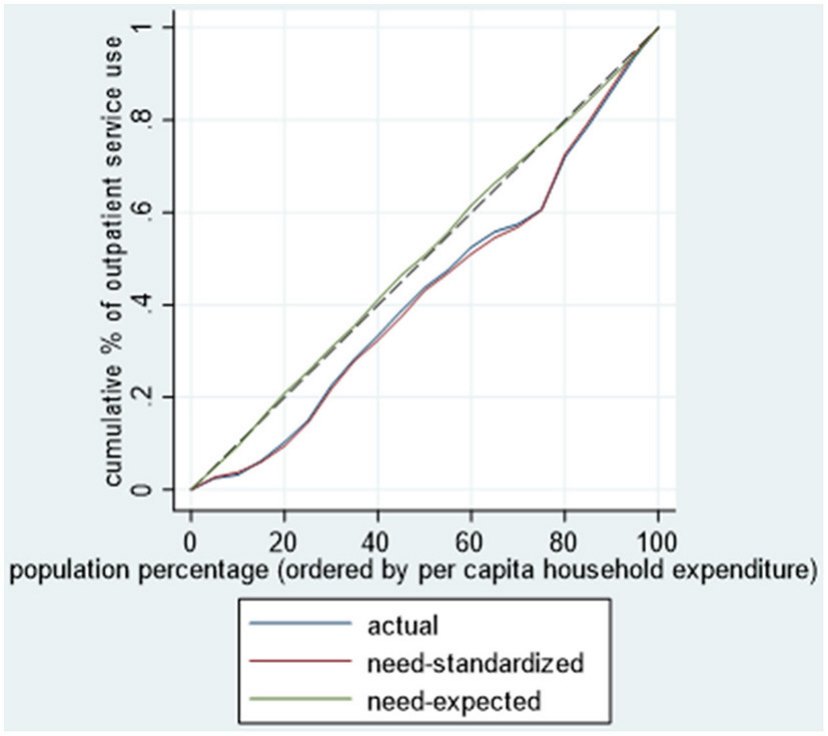
Concentration curve for use of outpatient visits among cancer patients.

**Figure 2:**
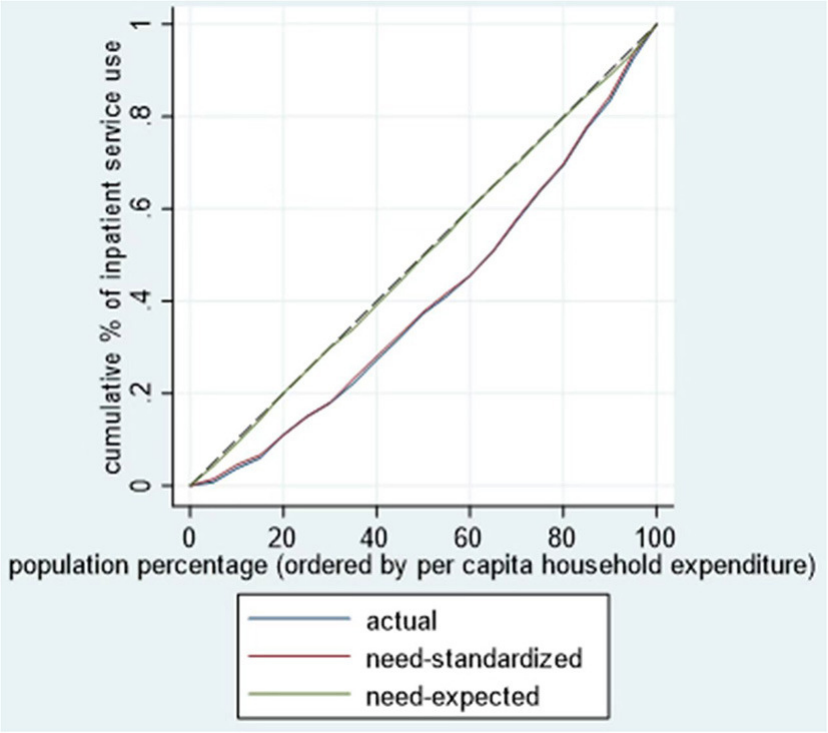
Concentration curve for use of inpatient services among cancer patients.

This study also revealed a 1.2-fold gap in outpatient visits use and a 1.3-fold gap in inpatient services use between the lowest income quintile and the highest income quintile after adjustment due to health needs. Indeed, after controlling for the distribution of needs, a significant pro-rich degree of inequality emerged (CI for outpatient visits = 0.1549, *p* < 0.05; CI for inpatient services = 0.1802, *p* < 0.05). As shown in Figures 1, 2, the concentration curves of actual and standardized outpatient and inpatient service use were all below the line of equality.

### Decomposition of inequality in cancer care use

Table 3 depicts the decomposition results and the contributions of various factors influencing the inequalities in cancer care use.

**Table 3:** Decomposition of socioeconomic-related inequalities in the use of outpatient and inpatient services among cancer patients.

**Variable**	**Outpatient visits use**			**Inpatient services use**		
	**Elasticity**	**CI**	**Contribution to CI (%)**	**Elasticity**	**CI**	**Contribution to CI (%)**
Gender (ref = male) female	0.7990	−0.0246	−13.86	−0.1245	−0.0246	1.56
**Age (ref** **=** **45–60, years)**						
60–75	0.1147	−0.0466	−3.77	0.0717	−0.0466	−1.71
≥75	0.0122	0.1163	1.00	0.0027	0.1163	0.16
**Educational level (ref** **=** **primary school or below)**						
Middle school	0.0126	0.1238	1.10	−0.1067	0.1239	−6.75
High school and above	0.1010	0.3724	26.49	0.0411	0.3723	7.79
Marital status [ref = single (separated/divorced/widowed/never married)] married or partnered	0.4027	0.0048	1.35	0.3012	0.0048	0.73
**Employment status (ref** **=** **unemployed)**						
Employed	−0.0196	−0.0133	18.36	−0.1097	−0.1329	7.44
Retired	−0.0396	0.3182	−8.88	−0.0437	0.3180	−7.08
Impoverished (ref = no) yes	0.0009	−0.0841	−0.05	0.0023	−0.0841	−0.10
**Region (ref** **=** **east)**						
Central	−0.0043	0.0388	−0.12	0.0514	0.0388	1.02
West	0.0208	−0.0472	−0.69	0.0419	−0.0472	−1.01
Northeast	0.0064	0.1224	0.55	0.0002	0.1224	0.01
**Hukou type (ref** **=** **agricultural Hukou)**						
Non-agricultural Hukou	0.0248	0.3021	5.28	−0.1673	0.3021	−25.78
**Region of residence (ref** **=** **urban area)**						
Suburban area	−0.0370	0.1825	−4.76	−0.0546	0.1825	−5.09
Rural village	0.0557	−0.1594	−6.26	−0.4246	−0.1594	34.53
The annual per capita household expenditure	1.9040	0.0589	78.99	2.7940	0.0589	83.92
**Health insurance (ref** **=** **no health insurance)**						
UEBMI	−0.1004	0.3137	−22.19	0.1373	0.3137	21.97
URRBMI	−0.0632	−0.1331	5.92	0.0449	−0.1331	−3.05
URBMI	−0.0210	0.1766	−2.62	−0.0063	0.1766	−0.57
NRCMS	−0.0494	−0.1120	3.90	0.1477	−0.1120	−8.44
Another	−0.0174	0.1862	−2.29	0.0074	0.1862	0.71
Number of people in the household	−0.1996	−0.0615	8.66	−0.0444	−0.0615	1.39
Physical examination (ref = no) yes	0.0817	0.1248	7.18	0.2452	0.1248	15.61
**Self-Reported health status (ref** **=** **very good)**						
Good	0.0239	−0.0764	−1.29	−0.0003	−0.0761	0.01
Fair	0.2856	−0.0879	−17.69	−0.0369	−0.0879	1.65
Poor	0.2502	0.0673	11.85	0.1367	0.0672	4.69
Very poor	0.1416	0.1069	10.66	0.0352	0.1069	1.92
Disability (ref = no) yes	0.0580	−0.0736	−3.00	0.1935	−0.0735	−7.25
**Pain degree (ref** **=** **none)**						
A little	0.0624	0.0247	1.01	0.1520	0.0248	1.92
Somewhat	0.1211	−0.0033	−0.28	0.0867	−0.0033	−0.14
Quite a bit	0.0228	0.0989	1.59	0.0561	0.0987	2.82
Very much	0.0744	−0.2036	−10.68	0.0486	−0.2036	−5.05
Smoking (ref = no) yes	0.2150	0.1312	19.89	0.1193	0.1312	7.99
Alcohol consumption (ref = no) yes	−0.0140	0.1157	−1.14	−0.0249	0.1157	1.47

Regardless of outpatient and inpatient services use, socioeconomic status made the greatest pro-rich contributions—that is, 78.99% and 83.92%, respectively, —followed by high school education (26.48% for outpatient services) and living in a rural village (34.53% for inpatient services). UEBMI made a great contribution to the pro-rich inequality in inpatient services (21.97%) while having a negative impact on outpatient visits (−22.19%). NRCMS had the opposite effect, but its contribution was relatively small. Among the need variables, a “health-poor” status (11.85%) and smoking (19.89%) had a positive contribution to the pro-rich inequality, while a “health-fair” status reduced the pro-rich inequality (−17.69% for outpatient services). The other variables provided relatively minor contributions to the inequity, as shown in Table 3.

## Discussion

Variations in the use of health care among cancer patients have attracted increased attention from both researchers and policymakers in related areas. To our knowledge, this is the first study to examine the association between socioeconomic status and health care use across different socioeconomic populations in China. Our study analyzed the distribution of the use of outpatient visits and inpatient services among patients with cancer from a perspective of equity. The analysis carried out here highlighted that cancer patients from higher socioeconomic status groups were more likely to use health care than those who were worse off. It was also evident that, after controlling for age, gender, and other need variables, there was a clear socioeconomic gradient in health care use. In addition, socioeconomic status and health insurance interacted to influence the risk of inequality in decomposition models.

In our study, the CIs for outpatient and inpatient services use were all positive, indicating that there was statistically significant inequality in the use of health care among cancer patients, in line with previous studies from South Korea and Australia (20, 21). Richer cancer patients appeared to be much more likely to use health care. In addition, this study revealed a greater extent of inequality compared to other research. A possible explanation may be that our study included individuals aged ≥45 years, and most incidence and deaths of cancer occurred in this age range (5). The health condition of these cancer patients might deteriorate due to inadequate sources of income (29), with the financial burden of age-related health rising (30). Compared to the entire population with cancer, the distribution of health care utilization among middle-aged and elderly cancer patients was more unequal.

Our study showed that higher inequality was generally in inpatient services in China. It could be explained by the fact that hospitalization costs were very high. Medical expenses (including medicines and treatments) and non-medical costs (including transportation, caregiver costs, lost productivity, and loss/reduction of household income) in inpatient services were higher than those in outpatient visits, which exacerbated the burden on health care use (31–33). Hence, cancer patients from socioeconomically disadvantaged households could not afford the high medical costs; actually, they tended to abandon medical services or sought cheaper outpatient services instead of inpatient services (34). Meanwhile, a lower socioeconomic status was related to a shorter survival time in cancer patients (11). Cancer patients with a higher socioeconomic status survived long enough to use additional inpatient services. Given this, inequalities in the utilization of inpatient services among cancer patients warrant more attention than disparities in outpatient visits.

We found that inequalities in health care among cancer patients remain largely determined by patients' financial capability in China. The key role of socioeconomic status in health care use was consistent with studies in other countries. Results of an Italy survey of individuals aged >50 years also indicated that income was a positive and significant determinant of use in preventive cancer care use (35). One possible explanation for this may be that, different from other diseases, cancer has more frequent recurrence, shorter disease-free survival, and higher mortality rates (1), placing a substantial economic burden on cancer sufferers and their families. Poor households were most likely to face impoverishment and economic hardship, entering a vicious circle of “poverty from illness and disease from poverty” (13, 36). Health care allocation and use are disproportionately favored by the better-off with higher education levels and, therefore, may widen inequalities further.

It is well-known that health insurance schemes are associated with health care use. Previous studies have shown that insured individuals were more likely to use health care than uninsured ones (37, 38). An incidence-based study that examined socioeconomic inequalities in Australia found that, apart from providing free medical services in public hospitals, Medicare had policies to protect patients from catastrophic health expenditures (31), defined as health-related out-of-pocket costs of ≥40% of total non-food household consumption expenditures (39). In our study, we observed UEBMI's pro-rich contributions to inpatient service use as well as the limited effects of URBMI and NRCMS, indicating that these health insurance schemes failed to protect low-income cancer patients, especially in terms of inpatient services (40). This result can be explained by certain reasons. First, although >96% of patients with cancer were covered by health insurance, UEBMI, URBMI, and NRCMS did not reimburse all medical services and items, especially expensive targeted therapies. Second, about 55.36% of participants with cancer in our study were unemployed, bearing the heavy burden of cancer therapy. In addition, these findings may be attributed to differences in the benefit packages between the different health insurance plans (41). UEBMI had a greater reimbursement rate than other health insurance schemes. The UEBMI beneficiaries were more willing to use expensive drugs and medical compared to the URBMI and NRCMS cancer patients (42). Evidence from an community-based study in China has confirmed that, in order to lessen the compensation gap between different insurances, the expansion of benefits packages should be tailored to differences between cancer patients in terms of income, health needs, and other factors (32).

UEBMI had different implications in outpatient and inpatient services on inequality. It could be explained that the cancer treatment choices varied in the different socioeconomic statuses. Due to the high cost of inpatient services, cancer patients with lower socioeconomic status were more willing to use outpatient services to alleviate, while surgical treatment was often chosen among the rich cancer patients (34). Therefore, for outpatient utilization, the disparities were relatively small. In addition, from the patients' socioeconomic status perspective, cancer patients who were covered by UEBMI were all urban workers or retired workers, they usually had higher income and better education compared to those with URBMI and NRCMS (43). Hence, they had a stronger incentive to utilize health care, which led to the significant effect on inequalities of UEBMI. UEBMI played a role in protecting the lower-income cancer patients from catastrophic health expenditure and had reduced financial burden in outpatient utilization, while cancer patients with higher socioeconomic status used more inpatient services, increasing the inequalities in inpatient utilization.

We did not find an apparent influence in health care use inequalities by age, although greater use by elderly individuals was observed. A possible reason for this result could be found in the sample characteristics, as only 13.52% of participants were aged ≥75 years. However, poverty, limited insurance coverage, education and awareness were factors that contributed to inequalities in cancer patients' health care use, in line with previous reports (32, 36). Wealth, the health insurance benefits package, and high school education increased the use of health care among cancer patients. Higher-income individuals had greater access to education, healthy dietary habits, and cancer care. This was also a good explanation for the pro-rich contribution of socioeconomic status to health care use among patients with cancer. Hence, a sustained reduction in socioeconomic inequalities concerning poverty would promote universal equality in health. In addition, more equitable and effective benefits packages committed to provide financial protection against catastrophic illness, such as expanding the public health insurance coverage of inpatient care to cancer patients, should also be designed.

Our study has some limitations. First, the diagnosis of cancer was self-reported, which might have led to under- or overestimation of the cancer prevalence. The information about health care use was also self-reported, so recall biases could not be avoided. In further research, more data sources and methods should be adopted to control these biases. Second, this study performed a cross-sectional analysis, which prevented us from discussing results based on causal inference. Third, the study sample might be not representative. Our sample size was relatively small and only included individuals aged ≥45 years. Fourth, since URBMI and NCMS have been merged, a comparison between UEBMI and URRBMI could be a better choice in future research (43). Finally, quality or efficiency measures should be included in inequality research; unfortunately, our survey did not provide relevant indicators (44).

## Conclusion

Significant differences were seen in the distribution of cancer care use across socioeconomic status groups in China, and a socioeconomic gradient was evident. Socioeconomic status and health insurance were found to be associated with inequalities. Interventions aimed at reducing inequalities in health care use should focus on improving financial protections for people from socioeconomically disadvantaged households.

## Data availability statement

The original contributions presented in the study are included in the article/supplementary material, further inquiries can be directed to the corresponding author/s.

## Author contributions

HZ led the study including the interpretation of the results and manuscript drafting. LS and MC conceived and supervised the study. YF contributed to data analysis and drafting of the manuscript. All authors reviewed and approved the final manuscript.

## Funding

This study was funded by the National Natural Science Foundation of China (Grant Nos. 71874086 and 72174093) and the Postgraduate Research & Practice Innovation Program of Jiangsu Province (Grant No. KYCX21_1561).

## Conflict of interest

The authors declare that the research was conducted in the absence of any commercial or financial relationships that could be construed as a potential conflict of interest.

## Publisher's note

All claims expressed in this article are solely those of the authors and do not necessarily represent those of their affiliated organizations, or those of the publisher, the editors and the reviewers. Any product that may be evaluated in this article, or claim that may be made by its manufacturer, is not guaranteed or endorsed by the publisher.

## References

[1] Leng A Jing J Nicholas S Wang J. Catastrophic health expenditure of cancer patients at the end-of-life: a retrospective observational study in China. BMC Palliat Care. (2019) 18:43. 10.1186/s12904-019-0426-5 31122235 PMC6533646

[2] Sung H Ferlay J Siegel RL Laversanne M Soerjomataram I Jemal A. Global cancer statistics 2020: GLOBOCAN estimates of incidence and mortality worldwide for 36 cancers in 185 countries. CA Cancer J Clin. (2021) 71:209–49. 10.3322/caac.21660 33538338

[3] Cao W Chen HD Yu YW Li N Chen WQ. Changing profiles of cancer burden worldwide and in China: a secondary analysis of the global cancer statistics 2020. Chin Med J. (2021) 134:783–91. 10.1097/CM9.0000000000001474 33734139 PMC8104205

[4] Qiu H Cao S Xu R. Cancer incidence, mortality, and burden in China: a time-trend analysis and comparison with the United States and United Kingdom based on the global epidemiological data released in 2020. Cancer Commun. (2021) 41:1037–48. 10.1002/cac2.12197 34288593 PMC8504144

[5] Chen W Zheng R Baade PD Zhang S Zeng H Bray F. Cancer statistics in China, 2015. CA Cancer J Clin. (2016) 66:115–32. 10.3322/caac.21338 26808342

[6] Anwar SL Adistyawan G Wulaningsih W Gutenbrunner C Nugraha B. Rehabilitation for cancer survivors: how we can reduce the healthcare service inequality in low- and middle-income countries. Am J Phys Med Rehabil. (2018) 97:764–71. 10.1097/PHM.0000000000000982 29905600

[7] Chen WQ Li H Sun KX Zheng RS Zhang SW Zeng HM. Report of cancer incidence and mortality in China, 2014. Zhonghua Zhong Liu Za Zhi. (2018) 40:5–13. 10.3760/cma.j.issn.0253-3766.2018.01.002 29365411

[8] The Global Health Data Exchange (GHDx) Database. Global Burden of Disease Study 2019 (GBD 2019) Data Resources. Available online at: https://vizhub.healthdata.org/gbd-results/ (accessed December 19, 2019).

[9] Hu G Mao A Dong P Yan X Qiu W. Discovery approach and economic burden of six kinds of common cancers patients in Beijing. Cancer Res Prev Treat. (2015) 42:171–6. 10.3971/j.issn.1000-8578.2015.02.016

[10] Li Y Xu J Gu Y Sun X Dong H Chen C. The disease and economic burdens of esophageal cancer in China from 2013 to 2030: dynamic cohort modeling study. JMIR Public Health Surveill. (2022) 8:e33191. 10.2196/33191 34963658 PMC8928052

[11] Yim J Hwang SS Yoo KY Kim CY. Contribution of income-related inequality and healthcare utilisation to survival in cancers of the lung, liver, stomach and colon. J Epidemiol Community Health. (2012) 66:37–40. 10.1136/jech.2009.104554 20961877

[12] Bernardes CM Whop LJ Garvey G Valery PC. Health service utilization by indigenous cancer patients in Queensland: a descriptive study. Int J Equity Health. (2012) 11:57. 10.1186/1475-9276-11-57 23051177 PMC3522530

[13] Norris RP Dew R Sharp L Greystoke A Rice S Johnell K. Are there socioeconomic inequalities in utilization of predictive biomarker tests and biological and precision therapies for cancer? A systematic review and meta-analysis. BMC Med. (2020) 18:282. 10.1186/s12916-020-01753-0 33092592 PMC7583194

[14] Paul C Boyes A Hall A Bisquera A Miller A O'Brien L. The impact of cancer diagnosis and treatment on employment, income, treatment decisions and financial assistance and their relationship to socioeconomic and disease factors. Support Care Cancer. (2016) 24:4739–46. 10.1007/s00520-016-3323-y 27364149

[15] Ozturk S Basar D. Equity in utilization of health care services in Turkey: an index based analysis. East Mediterr Health J. (2020) 26:547–55. 10.26719/emhj.19.090 32538448

[16] Bradley CJ Anderson-Mellies A Borrayo EA Doherty JA Escontrías OA Garcia DO. Ethnicity, socioeconomic status, income inequality, and colorectal cancer outcomes: evidence from the 4C2 collaboration. Cancer Causes Control. (2022) 33:533–46. 10.1007/s10552-021-01547-6 34982317

[17] Dalton SO Steding-Jessen M Gislum M Frederiksen K Engholm G Schüz J. Social inequality and incidence of and survival from cancer in a population-based study in Denmark, 1994-2003: background, aims, material and methods. Eur J Cancer. (2008) 44:1938–49. 10.1016/j.ejca.2008.06.010 18684615

[18] Parker L. Social inequality and cancer deaths. Lancet Oncol. (2001) 2:5. 10.1016/S1470-2045(00)00179-0 11905622

[19] Alleaume C Bendiane MK Peretti-Watel P Bouhnik AD. Inequality in income change among cancer survivors five years after diagnosis: evidence from a French national survey. PLoS ONE. (2019) 14:e0222832. 10.1371/journal.pone.0222832 31581224 PMC6776327

[20] Kim CW Lee SY Hong SC. Equity in utilization of cancer inpatient services by income classes. Health Policy. (2005) 72:187–200. 10.1016/j.healthpol.2004.03.009 15802154

[21] Fox P Boyce A. Cancer health inequality persists in regional and remote Australia. Med J Aust. (2014) 201:445–6. 10.5694/mja14.01217 25332023

[22] Maringe C Rachet B Lyratzopoulos G Rubio FJ. Persistent inequalities in unplanned hospitalisation among colon cancer patients across critical phases of their care pathway, England, 2011–13. Br J Cancer. (2018) 119:551–7. 10.1038/s41416-018-0170-2 30108292 PMC6162238

[23] Yoon TH Lee SY Kim CW Kim SY Jeong BG Park HK. Inequalities in medical care utilization by South Korean cancer patients according to income: a retrospective cohort study. Int J Health Serv. (2011) 41:51–66. 10.2190/HS.41.1.d 21319720

[24] Owen OD Eddy D Adam W Magnus L. Analyzing health Equity Using Household Survey Data: A Guide to Techniques and Their Implementation. Washington, DC: World Bank (2008).

[25] Wagstaff A Paci P van Doorslaer E. On the measurement of inequalities in health. Soc Sci Med. (1991) 33:545–57. 10.1016/0277-9536(91)90212-U 1962226

[26] Wagstaff A. Inequality aversion, health inequalities and health achievement. J Health Econ. (2002) 21:627–41. 10.1016/S0167-6296(02)00006-1 12146594

[27] Li YN Nong DX Wei B Feng QM Luo HY. The impact of predisposing, enabling, and need factors in utilization of health services among rural residents in Guangxi, China. BMC Health Serv Res. (2016) 16:592. 10.1186/s12913-016-1825-4 27760531 PMC5070132

[28] Wagstaff A Doorslaer Ev Watanabe N. On decomposing the causes of health sector inequalities with an application to malnutrition inequalities in Vietnam. J Econom. (2003) 112:207–23. 10.1016/S0304-4076(02)00161-6

[29] Li C Dou L Wang H Jing S Yin A. Horizontal inequity in health care utilization among the middle-aged and elderly in China. Int J Environ Res Public Health. (2017) 14:842. 10.3390/ijerph14080842 28933772 PMC5580546

[30] Hou Y Dan X Babbar M Wei Y Hasselbalch SG Croteau DL. Ageing as a risk factor for neurodegenerative disease. Nat Rev Neurol. (2019) 15:565–81. 10.1038/s41582-019-0244-7 31501588

[31] Mahumud RA Alam K Dunn J Gow J. Emerging cancer incidence, mortality, hospitalisation and associated burden among Australian cancer patients, 1982–2014: an incidence-based approach in terms of trends, determinants and inequality. BMJ Open. (2019) 9:e031874. 10.1136/bmjopen-2019-031874 31843834 PMC6924826

[32] Mao Y Xu F Zhang MJ Liu JL Yang J Wang MJ. Equity of health service utilization of urban residents: data from a western Chinese city. Chin Med J. (2013) 126:2510–6. 10.3760/cma.j.issn.0366-6999.20123001 23823826

[33] Guo B Xie X Wu Q Zhang X Cheng H Tao S. Inequality in the health services utilization in rural and urban China: a horizontal inequality analysis. Medicine. (2020) 99:e18625. 10.1097/MD.0000000000018625 31914043 PMC6959938

[34] Røe OD. The high cost of new cancer therapies—a challenge of inequality for all countries. JAMA Oncol. (2017) 3:1169–70. 10.1001/jamaoncol.2016.6335 28033441

[35] Carrieri V Wuebker A. Assessing inequalities in preventive care use in Europe. Health Policy. (2013) 113:247–57. 10.1016/j.healthpol.2013.09.014 24409498

[36] Liu Y Liu N Cheng M Peng X Huang J Ma J. The changes in socioeconomic inequalities and inequities in health services utilization among patients with hypertension in Pearl River Delta of China, 2015 and 2019. BMC Public Health. (2021) 21:903. 10.1186/s12889-021-10879-6 33980187 PMC8117279

[37] Zhu D Guo N Wang J Nicholas S Chen L. Socioeconomic inequalities of outpatient and inpatient service utilization in China: personal and regional perspectives. Int J Equity Health. (2017) 16:210. 10.1186/s12939-017-0706-8 29202843 PMC5715559

[38] Garcia-Ramirez J Nikoloski Z Mossialos E. Inequality in healthcare use among older people in Colombia. Int J Equity Health. (2020) 19:168. 10.1186/s12939-020-01241-0 33100214 PMC7646194

[39] Cylus J Thomson S Evetovits T. Catastrophic health spending in Europe: equity and policy implications of different calculation methods. Bull World Health Organ. (2018) 96:599–609. 10.2471/BLT.18.209031 30262941 PMC6154073

[40] Kim S Kwon S. The effect of extension of benefit coverage for cancer patients on health care utilization across different income groups in South Korea. Int J Health Care Finance Econ. (2014) 14:161–77. 10.1007/s10754-014-9144-y 24691773

[41] Xie X Wu Q Hao Y Yin H Fu W Ning N. Identifying determinants of socioeconomic inequality in health service utilization among patients with chronic non-communicable diseases in China. PLoS ONE. (2014) 9:e100231. 10.1371/journal.pone.0100231 24960168 PMC4069022

[42] Li Z Hung P He R Tu X Li X Xu C. Disparities in end-of-life care, expenditures, and place of death by health insurance among cancer patients in China: a population-based, retrospective study. BMC Public Health. (2020) 20:1354. 10.1186/s12889-020-09463-1 32887583 PMC7650520

[43] Li Y Yang Y Yuan J Huang L Ma Y Shi X. Differences in medical costs among urban lung cancer patients with different health insurance schemes: a retrospective study. BMC Health Serv Res. (2022) 22:612. 10.1186/s12913-022-07957-9 35524258 PMC9077891

[44] Fu XZ Wang LK Sun CQ Wang DD He JJ Tang QX. Inequity in inpatient services utilization: a longitudinal comparative analysis of middle-aged and elderly patients with the chronic non-communicable diseases in China. Int J Equity Health. (2020) 19:6. 10.1186/s12939-019-1117-9 31906960 PMC6945393

